# Case report: A case of complete resolution of obstructive and central sleep apnea with Cheyne Stokes breathing in a patient with heart failure 60 days post-left ventricular assist device implantation

**DOI:** 10.3389/frsle.2023.1228038

**Published:** 2023-09-07

**Authors:** Saif Mashaqi, Michael William, Stuart F. Quan, Daniel Combs, Lauren Estep, Salma I. Patel, Jyotsna Sahni, Sairam Parthasarathy

**Affiliations:** ^1^Department of Pulmonary, Allergy, Critical Care and Sleep Medicine, The University of Arizona College of Medicine, Tucson, AZ, United States; ^2^Division of Sleep and Circadian Disorders, Division of Sleep Medicine, Brigham and Women's Hospital, Harvard Medical School, Boston, MA, United States; ^3^Division of Pulmonary and Sleep Medicine, Department of Pediatrics, UAHS Center for Sleep and Circadian Sciences, University of Arizona, Tucson, AZ, United States; ^4^Department of Sleep Medicine, Swan Sleep Medicine, Tucson, AZ, United States

**Keywords:** central sleep apnea, Cheyne Stokes breathing, left ventricular assist device, heart failure, obstructive sleep apnea

## Abstract

Sleep-disordered breathing (obstructive and central sleep apnea) are common in patients with heart failure with reduced ejection fraction. Herein, we report a 69-year-old patient with a history of severe heart failure and refractory ventricular arrhythmia who was diagnosed with a moderate degree of obstructive and central sleep apnea with Cheyne Stokes breathing. He underwent a successful implantation of left ventricular assist device. Our patient had a complete resolution of both obstructive and central sleep apnea 60 days post-LVAD implantation as confirmed by home sleep apnea test.

## Introduction

Heart failure (HF) remains one of the common cardiovascular diseases in the US and worldwide. Although the incidence of HF remains the same since 2010, the burden of hospitalization and mortality continue to rise (Roger, 2021). Sleep-disordered breathing (SDB) including both obstructive and central sleep apnea are common in HF. Central sleep apnea (CSA), in particular, is highly prevalent (Ferrier et al., 2005) and carries an adverse prognostic value since it is associated with higher hospital readmission rates (Khayat et al., 2012).

In patients with systolic heart failure and reduced ejection fraction (HFrEF), a special form of CSA can be encountered called Hunter-Cheyne-Stoke breathing (CSB). It is characterized by ≥ three consecutive central apneas and/or hypopneas separated by a crescendo and decrescendo change in breathing amplitude with a cycle length ≥ 40 s and ≥ 5 central apneas and/or central hypopneas per hour of sleep associated with the crescendo/decrescendo breathing pattern over ≥ 2 h of monitoring (Berry et al., 2012).

Although several treatment options are available for CSA-CSB in the setting of HFrEF [such as positive air way pressure (PAP), positional therapy, medications, or phrenic nerve stimulation (Wang et al., 2023)], the first line is always to optimize cardiac function. We report in this manuscript a successful resolution of both obstructive and central sleep apnea post left ventricular assist device implantation in a patient with severe HFrEF.

## Case description

We report a 69-year-old white Caucasian male with history of non-ischemic cardiomyopathy with reduced ejection fraction (EF ~15–20%), complicated by refractory non-sustained ventricular tachycardia. He presented to sleep clinic complaining of fatigue, loud snoring, witnessed apnea, choking, and gasping as well as recurrent awakenings during the night secondary to nocturia and sometimes for unknown reasons. Epworth Sleepiness Scale (ESS) was 3/24. On physical examination, his blood pressure was 117/67 mmHg and pulse rate 68 per min. His body mass index was 26.7 kg/m^2^, Mallampati score was III/IV, and neck circumference was 40 cm. There was no retrognathia, the rest of the physical examination was within normal limits.

**Figure 1:**
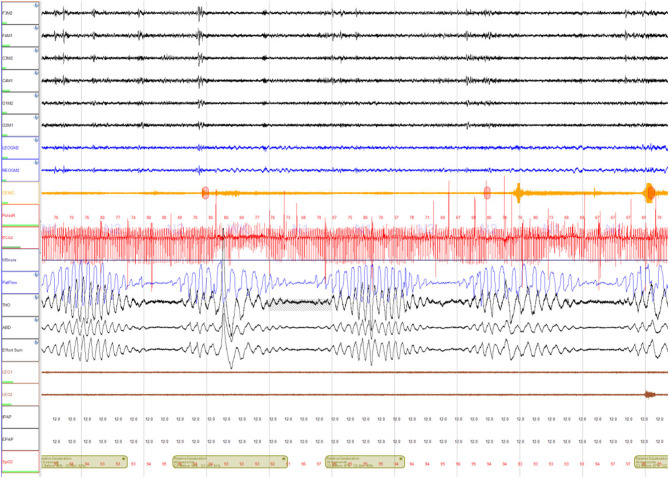
A 5-min epoch demonstrating central sleep apnea with Cheyne Stokes breathing (CSA-CSB).

He underwent a home sleep apnea test (HSAT) which showed a moderate degree of obstructive sleep apnea (OSA) (respiratory event index (REI) of 22 events per hour, oxygen desaturation index (ODI) OF 33.5 events per hour, mean SpO_2_ 90%, nadir SpO_2_ 83%, and time spent with SpO_2_ <88% (T-88%) 1 h and 36 min). This study was followed by an unacceptable full night continuous positive (CPAP) airway pressure titration study. CPAP settings of 4 cm H_2_O to 13 cm H_2_O were tested, however, none of these settings normalized the apnea-hypopnea index. The overall AHI during the study was 19 events per hour (central AHI 10 events per hour and obstructive AHI 9 events per hour). Most of the central events resembled CSB (Figure 1). He was scheduled for a full night Bi Level PAP titration study, but unfortunately, the patient was admitted to the hospital for acute decompensated congestive heart failure.

The patient has a complicated cardiac history, including HFrEF with EF ~20% with recurrent sustained ventricular tachycardia/ventricular fibrillation status post Bi Ventricular Implantable Cardioverter Defibrillator (ICD), atrial fibrillation/flutter status post ablation, and moderate mitral regurgitation. During hospital admission, he underwent left heart catheterization which showed normal coronary arteries followed by right heart catheterization that showed severe pre- and post- capillary pulmonary hypertension [The initial hemodynamic data from the pulmonary artery (PA) catheter were: right atrial (RA) pressure 18 mmHg, right ventricle (RV) systolic pressure 72 mmHg, RV diastolic pressure 18 mmHg, PA systolic pressure 75 mmHg, PA diastolic pressure 40 mmHg, PA mean pressure 52 mmHg, pulmonary capillary wedge pressure (PCWP) 40 mmHg, pulmonary vascular resistance (PVR) 3.8 dyne/cm^2^, systemic vascular resistance (SVR) 1981 dyne/cm^2^, and cardiac index (CI) 1.5 l/min/m^2^]. While in the cardiovascular intensive care unit (CVICU) for close hemodynamic monitoring, he had several runs of non-sustained ventricular tachycardia (NSVT) and ventricular tachycardia (VT) and ICD fired appropriately despite the use of mexiletine.

**Figure 2:**
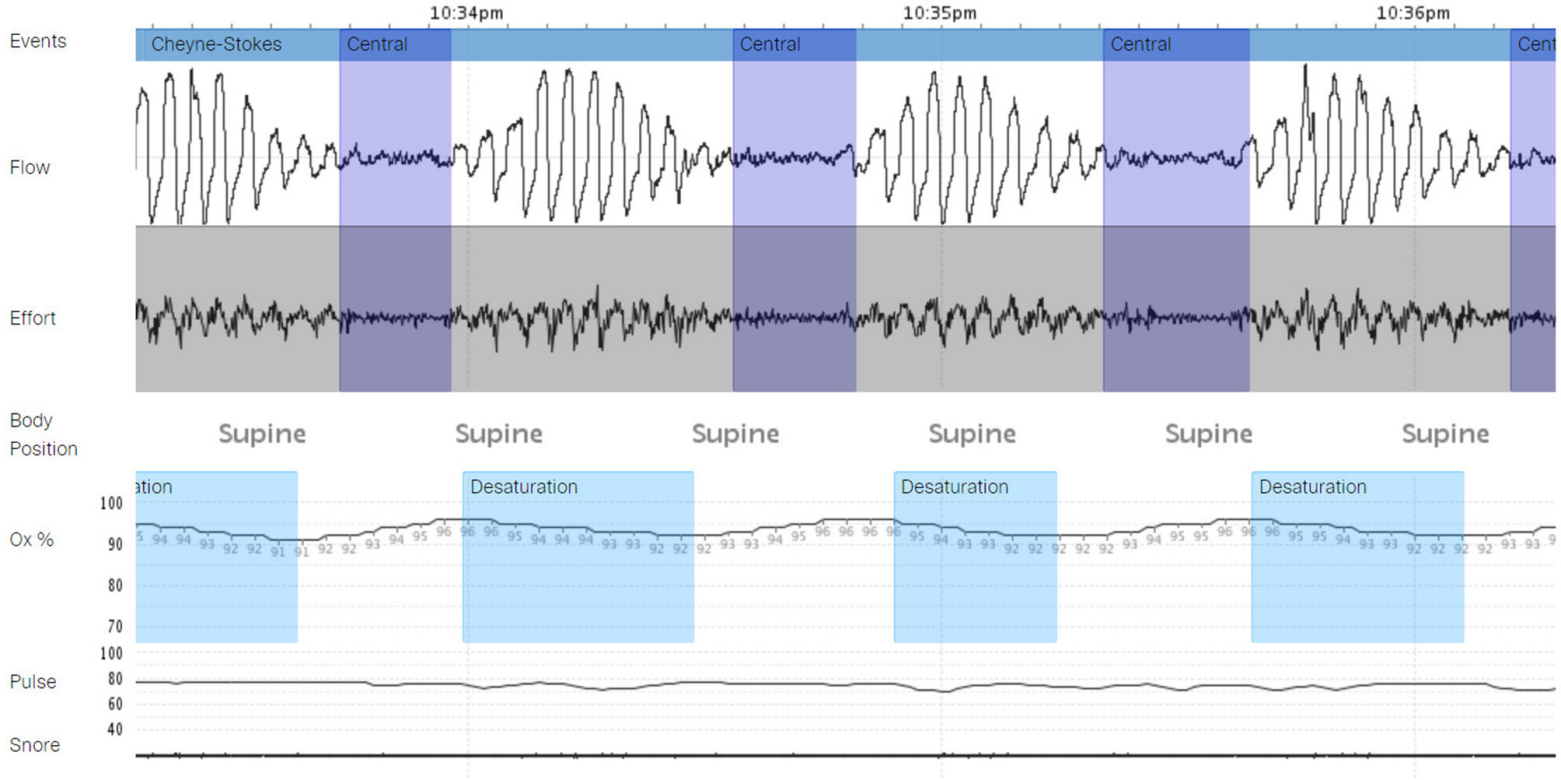
A portable monitor test demonstrating central sleep apnea with Cheyne Stokes breathing (CSA-CSB) – test conducted in the hospital before left ventricular assist device (LVAD) implantation.

**Figure 3:**
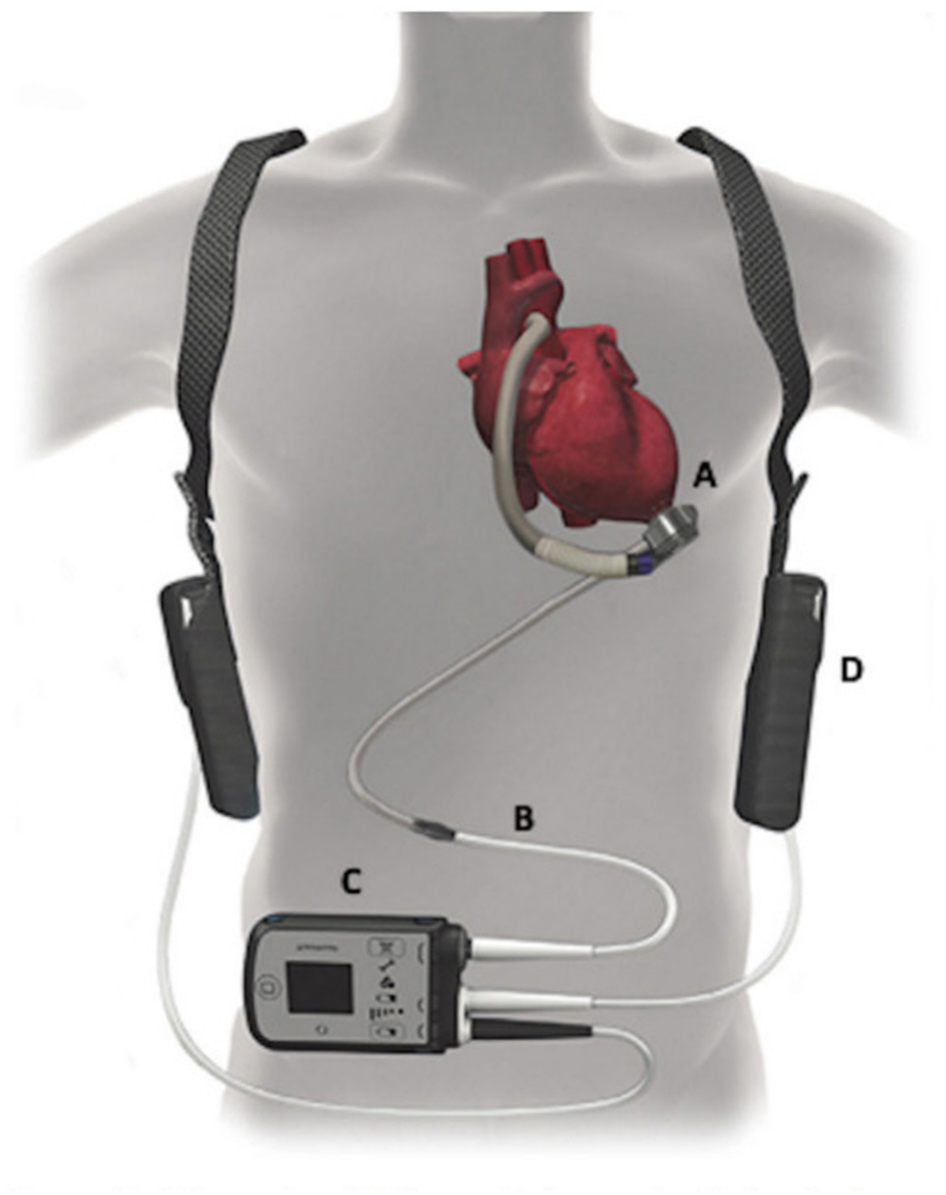
The HeartMate 3 left ventricular assist device (LVAD) system - Abbott cardiovascular. Reproduced from DeFilippis et al. (2019) with permission from John Wiley and Sons.

The patient declined listing for a heart transplant. Alternatively, he opted to proceed with left ventricular assist device (LVAD) implantation. During the preparation for surgery, several nurses noticed an association between the timing of VT and apnea, gasping, and choking. Sleep Medicine was consulted, and a portable monitor sleep apnea test [Type III ApneaLink air device, (ResMed, San Diego, CA)] was conducted 3 days before LVAD implantation which confirmed a moderate degree of obstructive and central sleep apnea (REI 17 events per hour, central REI 5 events per hour). Central sleep apnea resembled CSB (Figure 2). He was started empirically on Bi Level PAP (IPAP 10 cm H_2_O and EPAP 5 cm H_2_O). Three days later, the patient underwent implantation of HeartMate 3 LVAD (Abbott cardiovascular Plymouth, MN 55442) (Figure 3) with the following settings (flow: 3.9 LPM, speed: 4800 rpm, P.I. 3.0, power: 3.3 watts). Hemodynamics numbers improved after LVAD (PA systolic pressure 35 mmHg, PA diastolic pressure 14 mmHg, PA mean pressure 21 mmHg, PVR 2 dyne/cm^2^, SVR 1251 dyne/cm^2^, CI 1.8 l/min/m^2^). He was discharged from the hospital 4 weeks later.

**Figure 4:**
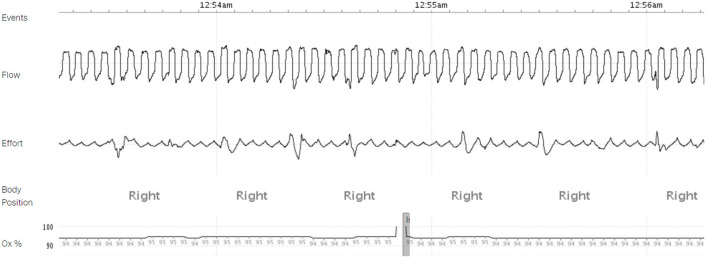
A home sleep apnea test (HSAT) demonstrating resolution of obstructive and central sleep apnea 60 days post-VAD implantation.

Six weeks later, he was evaluated in the sleep clinic. His wife mentioned near resolution of the snoring, apnea, gasping, and choking with fewer episodes of waking after sleep onset. A follow up HSAT conducted 2 months after LVAD implantation showed a normal REI of 1.3 events per hour with complete resolution of central and obstructive sleep apnea, ODI 0.0 events per hour, mean SpO_2_ 94%, nadir SpO_2_ 86%, and T-88% 10 min (Figure 4).

## Discussion

The effect of LVAD on SDB has not been systemically investigated and available data is limited to case reports and case series. The first case series was published by Padeletti et al. (2007) where three patients with NYHA class IV, EF ~15–20% (non-ischemic cardiomyopathy), and moderate to severe CSA with CSB and no OSA underwent LVAD implantation as a bridge to heart transplant but unfortunately, CSA/CSB did not resolve. The follow up polysomnogram (PSG) after LVAD implantation was done <30 days afterwards in 2/3 patients and 2/3 patients had a severely dilated right ventricle and elevated right ventricular systolic pressure. In contrast, Vazir et al. (2010) reported a resolution of CSA/CSB post-LVAD implant in a 62-year-old patient with milder congestive heart failure symptoms (NYHA class III, EF ~27%) who presented with severe CSA with CSB and no OSA. However, the repeat PSG was conducted 10 months post-VAD implant. Vermes et al. (2009) had a similar case with CSA/CSB but with associated OSA that responded very well to LVAD implantation. Both CSA and OSA resolved, and the patient underwent heart transplant 2 months after VAD implant. Interestingly, a repeat PSG 1 year later, showed recurrence of OSA (no CSA) which was most likely related to weight gain. Kumai et al. (2022) studied a cohort of 50 patients who underwent HeartMate II (HMII; Abbott, Lake Bluf, IL, USA) and HSAT and concluded that SDB prevalence was 24% in this cohort. These patients have been found to have a higher risk of ventricular tachyarrhythmias compared to the non-SDB group (Chowdhury et al., 2017).

Why is there persistence of CSA-CSB in some cases post-VAD implantation and even post-transplant (Thalhofer et al., 2000)? This suggests that there are other factors (non-cardiac) that might contribute to CSA-CSB. One such factor relates to the impact of loop gain which promotes the occurrence of CSA-CSB. High loop gain increases the tendency for persistent CSA-CSB while low loop gain mitigates it (Sands and Owens, 2015). The magnitude of loop gain is represented by the following equation.


$$\begin{array}{l} Loopgain=G^{*}PaCO2^{*}T/Lungvolume \end{array}$$

(G = constant represents the chemoreceptor sensitivity, T= circulation time that reflects EF)

Circulatory time delay secondary to reduced EF is only one factor in the equation. In fact, chemoreceptor sensitivity is believed to be one of the powerful factors influencing the loop gain. Other factors include lung volume, mainly functional residual capacity (FRC). Pulmonary diseases that decrease FRC such as pulmonary edema and pleural effusion tend to increase loop gain. To further understand the loop gain concept and each element mentioned in the above equation, it is important to focus briefly on the three classic components of loop gain that reflect the ventilatory response to a disturbance. (1) The controller gain which reflects the degree to which minute ventilation increases for a given disturbance. This is represented by the chemoreceptor sensitivity. (2) The plant gain which reflects the magnitude of the change in arterial gas in response to a change in minute ventilation. This is represented by the respiratory system and lung volumes. (3) The mixing gain which represents the transfer of information (i.e., the circulation time and pulmonary capillary changes) (Javaheri and Badr, 2023).

It is interesting to see complete resolution of OSA in our patient after VAD implantation despite the minimal change in BMI. One possible explanation is a reversal in the overnight rostral fluid shift in upper airway which has been shown to worsen OSA. This can be reversed by physical activity (Redolfi et al., 2015). It is possible that increased physical activity after VAD implantation resolved the OSA in our patient. Furthermore, there might be a role for BNP levels as a predictor for the risk of SDB 60 days post-VAD implantation with a cut-of value of 322 pg/mL (Kumai et al., 2022). Unfortunately, our patient did not have BNP level measured at that time.

The main limitation in our case report is that we did not conduct an in-lab PSG after VAD implantation. This was discussed with the patient, and he elected to do a HSAT. Use of HSAT in patients with VAD has not been systemically evaluated (Akkanti et al., 2017).

## Conclusion

We report a complete resolution of moderate OSA and CSA-CSB after <60 days of LVAD implantation in a patient with severe HFrEF. A long-term follow-up (1 year) is warranted to re-evaluate the risk of recurrence of OSA and CSA-CSB.

## Patient perspective

The patient was extremely satisfied with the impact of LVAD implant on his sleep. He said “I don't wake up very frequently as before, I still wake up to urinate, but I have prostate problems. It is rewarding to feel more alert during the daytime”. His wife added also “I don't witness anymore the loud snoring and the scary apnea episodes that he used to have which helped with my sleep”.

## Data availability statement

The raw data supporting the conclusions of this article will be made available by the authors, without undue reservation.

## Ethics statement

Written informed consent was obtained from the individual(s) for the publication of any potentially identifiable images or data included in this article.

## Author contributions

SM contributed to the conception of the manuscript. MW, DC, SIP, SQ, LE, SP, and JS reviewed the manuscript. SM wrote the first draft of the manuscript. MW wrote sections of the manuscript. SM, MW, and JS interviewed the patient, took history and physical examinations, and interpreted his sleep studies. All authors contributed to manuscript revision, read, and approved the submitted version.
